# Synthesis of Polymer Brushes on Tannic Acid-Coated Copper Particles and Surface Co-Assembly

**DOI:** 10.3390/polym16111587

**Published:** 2024-06-03

**Authors:** Chen Wang, Hanying Zhao

**Affiliations:** Key Laboratory of Functional Polymer Materials of the Ministry of Education, College of Chemistry, Nankai University, Tianjin 300071, China; 2120170821@mail.nankai.edu.cn

**Keywords:** copper particles, polymer brushes, surface nanostructures, surface co-assembly, hollow capsules

## Abstract

The synthesis of polymer brushes on inorganic particles is an effective approach to surface modification. The polymer brushes on the surface endow the substrates with new surface properties. However, the lack of functional groups and the difficulty of surface modification have made it difficult to develop an effective method for the synthesis of polymer brushes on metal surfaces. Herein, a simple and versatile strategy for synthesizing polymer brushes on copper particles is reported. Tannic acid (TA) molecules are adsorbed onto the surfaces of copper particles, forming TA coatings. Quaternized poly(2-(dimethylamino)ethyl methacrylate)-*block*-polystyrene (qPDMAEMA-*b*-PS) block copolymer (BCP) chains are grafted on the TA coatings through hydrogen bonding and electrostatic interaction, and PS brushes are grafted on the copper particles. The effects of TA concentration on the adsorption of TA and PS brush synthesis are discussed. The PS brushes are able to form surface nanostructures on the copper particles through co-assembly with PDMAEMA-*b*-PS BCP chains. The effect of BCP concentration on the surface nanostructures is investigated. It is reasonable to expect that polymer brushes and surface nanostructures can be synthesized on different metal surfaces by using the TA-coating approach reported in this paper.

## 1. Introduction

The grafting of polymer brushes onto surfaces is an effective approach to the surface modification of inorganic particles [1,2,3,4,5]. Many properties of the particles, especially the dispersion and the stability of the particles in organic solvents, have been improved due to the synthesis of the polymer brushes on the surfaces. Polymer brushes refer to the polymer chains that are tethered to the solid surfaces at high grafting densities [6,7,8]. Polymer brushes can be synthesized by using the “grafting to” or “grafting from” methods [9,10,11,12]. In the “grafting to” approach, polymer chains are grafted to the surfaces at chain ends through chemical reactions or specific interactions between polymer chains and particle surfaces [13,14,15,16]. In the “grafting from” approach, polymer chains grow from the initiator or chain transfer agent (CTA)-modified solid surfaces [17,18,19,20,21].

In both the “grafting to” and “grafting from” methods, it is necessary to achieve surface functionalization in order to introduce reactive functional groups or initiator (or CTA) groups onto the surfaces. In the past few decades, many studies on surface functionalization have been conducted. In order to synthesize polymer brushes through click chemistry, azide groups (or alkynes) have been introduced onto the surfaces of substrates [22,23]. Thiol groups or pyridyl disulfide groups were prepared on the surfaces of particles, and polymer brushes on the particle surfaces were prepared through thiol chemistry [24,25,26,27,28,29,30]. Atom transfer radical polymerization (ATRP) initiators or reversible addition–fragmentation chain transfer (RAFT) CTAs were introduced onto the solid surfaces and polymer brushes were prepared by using the “grafting from” approach [31,32].

**Scheme 1:**
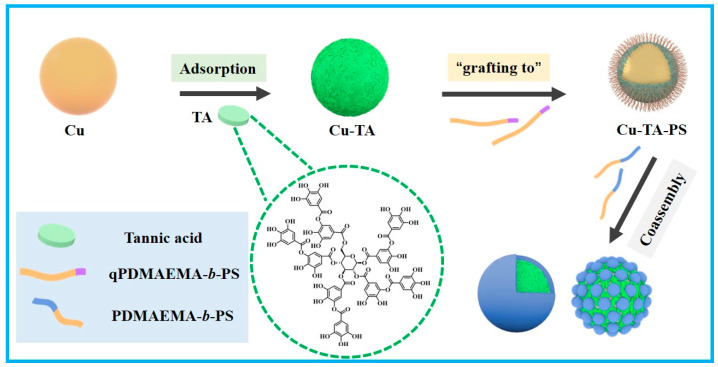
A schematic illustration of the coating of copper particles with TA, the synthesis of PS brushes on copper particles by using the “grafting to” approach and the fabrication of the surface nanostructures by using the co-assembly approach.

Although great success has been achieved in the synthesis of polymer brushes, it is difficult to synthesize polymer brushes on metal particles due to the difficulty of surface functionalization. TA contains a large number of phenolic hydroxyl groups in its chemical structure which have strong hydrogen bond interactions with amino groups. The phenol hydroxyl anions have strong electrostatic interactions with the positively charged quaternized polymer blocks [33,34]. Previously, it has been demonstrated that tannic acid (TA) has a high surface binding affinity to a wide range of material surfaces due to the catechol/pyrogallol groups on the structures. TA has been used as building blocks in the fabrication of multilayer films and hollow capsules [35]. In a previous concept study, TA was coated on the surface of silica particles, and PS brushes were prepared on the TA coatings [36]. In that approach, surface modification of the substrate was not necessary as the TA-coating approach provides a direct, simple and versatile method for the fabrication of polymer brushes on substrate surfaces. In this study, we demonstrate that polymer brushes can be fabricated on the surface of copper particles by using the TA-coating method and nanostructures are constructed through surface co-assembly with a copolymer. As shown in Scheme 1, TA is adsorbed onto the surface of copper particles through noncovalent-specific interactions (Cu-TA). The TA coatings are used as a platform for the synthesis of polymer brushes. Partially quaternized poly(2-(dimethylamino)ethyl methacrylate)-*block*-polystyrene (qPDMAEMA-*b*-PS) block copolymer (BCP) chains are grafted onto the TA coatings through hydrogen bonding and electrostatic interactions, resulting in the synthesis of PS brushes on TA coatings (Cu-TA-PS). Due to the grafted hydrophobic polymer brushes on the metal surface, it is reasonable to expect that this technology will be applied for metal anti-corrosion [37,38,39]. Hollow capsules are obtained after etching copper particles with hydrochloric acid. It can be used as a drug storage space or a nanoreactor and has potential application value in the fields of drug-controlled release, biological energy storage and nano-catalysis.

## 2. Materials and Methods

### 2.1. Materials

Styrene (98%, Tianjin Chemical reagent Company, Tianjin, China) and DMAEMA (98%, Aldrich) were purified by being passed through basic Al_2_O_3_ columns and distilled under reduced pressure. 2,2′-Azoisobutyronitrile (AIBN, 98%, Guo Yao Chemicals) was recrystallized in ethanol and dried under reduced pressure. TA was purchased from Aldrich and used as received. The synthesis of 4-cyanopentanoic acid dithiobenzoate (CPADB) was reported in our previous publication [29]. Spherical copper particles with an average size of 400 nm were purchased from Macklin and used as received. All of the solvents used in this study were purified before use.

### 2.2. Synthesis of PDMAEMA-b-PS

PDMAEMA-*b*-PS was synthesized through sequential RAFT polymerization. PDMAEMA-CTA was prepared through RAFT polymerization of DMAEMA using CPADB as a chain transfer reagent. The typical procedure is described as follows. CPADB (223 mg, 0.800 mmol), DMAEMA (6.2 g, 40 mmol) and AIBN (19.7 mg, 0.120 mmol) were dissolved in 10 mL of 1,4-dioxane in a 50 mL Schlenk flask. RAFT polymerization of DMAEMA was carried out at 70 °C for 6 h after three freeze–pump–thaw cycles. PDMAEMA-CTA was obtained by precipitating it into a 10-fold excess of hexane and drying it under reduced pressure at room temperature. PDMAEMA-*b*-PS was prepared through RAFT polymerization of styrene using PDMAEMA-CTA as a macromolecular chain transfer reagent. Styrene (2.77 g, 26.6 mmol), PDMAEMA-CTA (200 mg, 0.0440 mmol) and AIBN (1.0 mg, 0.0060 mmol) were dissolved in 2.5 mL of toluene in a 10 mL Schlenk flask. RAFT polymerization of styrene was carried out at 90 °C for 12 h after three freeze–pump–thaw cycles. PDMAEMA-*b*-PS was obtained by precipitating it into a 10-fold excess of hexane and drying it under reduced pressure at room temperature.

### 2.3. Synthesis of TA-Modified Copper Particles (Cu-TAs)

Copper particles (10 mg) were dispersed in 1 mL of a TA aqueous solution at a concentration of 0.1 mg/mL, and the mixture was stirred at room temperature for 10 h. After being washed with ultrapure water and THF, Cu-TAs were collected and dried under reduced pressure. The amount of TA adsorbed on copper particles was determined by UV–vis.

### 2.4. Synthesis of Polymer Brushes on Cu-TA (Cu-TA-PS)

There were two steps in the synthesis of polymer brushes on Cu-TA. In the first step, PDMAEMA_27_-*b*-PS_130_ BCPs were quaternized with methyl iodide; and in the second step, polymer brushes on Cu-TA were synthesized by anchoring the quaternized BCP chains onto the surfaces of Cu-TA through electrostatic interactions and hydrogen bonding. The typical procedures are described as follows. PDMAEMA_27_-*b*-PS_130_ (20 mg) was dissolved in 20 mL of DMF in a 50 mL flask, 20 µL of iodomethane was dissolved in 1.98 mL of DMF (0.01 µL/mL) and 94 μL of the solution was added to the polymer solution. A quaternization reaction was performed at 40 °C in the dark for 24 h. After the reaction, partially quaternized BCPs (qPDMAEMA_27_-*b*-PS_130_) were obtained. Cu-TA (50 mg) was added to the polymer solution under ultrasonication, and the mixture was stirred at room temperature for 12 h. After the adsorption, copper particles with polymer brushes on the surfaces were collected through centrifugation. After being washed with DMF five times, copper particles with polymer brushes (Cu-TA-PS) were dried under reduced pressure.

### 2.5. Co-Assembly of Cu-TA-PS and PDMAEMA-b-PS

A typical surface co-assembly procedure is described as follows. Cu-TA-PS (5 mg) and PDMAEMA_50_-*b*-PS_136_ (1.0 mg) were dispersed/dissolved in 0.5 mL of THF. Methanol (3.5 mL) was added to the mixture at a flow rate of 0.1 mL/min. After surface co-assembly, the copper particles were washed with methanol three times and dried under reduced pressure. The hybrid particles with surface nanostructures (Cu-TA-PS/PDMAEMA-*b*-PS) were collected.

### 2.6. Fabrication of Hollow Capsules

Hollow capsules were prepared by etching copper particles with a HCl aqueous solution. In a flask, Cu-TA-PS/PDMAEMA-*b*-PS (5 mg) was dispersed in 1 mL of a HCl aqueous solution (pH = 1) and the solution was stirred at room temperature for 12 h. After the reaction, hollow capsules were collected through centrifugation and washed with methanol.

### 2.7. Characterization

^1^H NMR measurements were performed on a Bruker Avance III 400 MHz spectrometer using deuterated chloroform as the solvent. The apparent molecular weight and distribution of BCPs were characterized by using size exclusion chromatography. The details about the equipment can be found in our previous publication [29]. The ultraviolet absorption spectrum was collected on a Shimadzu UV-2450 spectrophotometer with a wavelength of 190–800 nm. Quartz cuvettes with a width of 2 nm were used. The surface nanostructures on copper particles were analyzed under the Tecnai G2 F20 S-TWIN electron microscope operating at 200 kV. The TEM specimens were prepared by depositing diluted solutions of the samples on carbon-coated copper grids and evaporating the solvent at room temperature. SEM images of the copper particles were obtained by using the Apreo S LoVac Thermo Scientific scanning electron microscope. SEM specimens were prepared by depositing diluted solutions of the samples on a silicon wafer and evaporating the solvent at room temperature. The zeta potential values of the copper particles were obtained on a Malvern Zetasizer Nano-ZS equipped with a 633 nm He-Ne laser. Thermogravimetric analysis (TGA) curves were obtained by using a Synchronous Thermal Analyzer (STA 499 F5). Before measurements, all of the samples were dried at 70 °C for 12 h and at room temperature for 24 h under reduced pressure. All of the samples were heated to 800 °C at a heating rate of 10 K/min under nitrogen atmosphere. The data of TGA are normalized at 100 °C.

## 3. Results

### 3.1. Synthesis of Polymer Brushes on TA-Coated Copper Particles (Cu-TA-PSs)

PDMAEMA-*b*-PS BCPs were synthesized through RAFT polymerization. The average repeating unit numbers of PDMAEMA and PS blocks were determined by ^1^H NMR. Two BCPs with different PDMAEMA block lengths and a similar PS block length, PDMAEMA_50_-*b*-PS_136_ and PDMAEMA_27_-*b*-PS_130_, were synthesized in this study. The ^1^H NMR result and size exclusion chromatography (SEC) curves of the BCPs are shown in Figure S1. The quaternized PDMAEMA_27_-*b*-PS_130_ (qPDMAEMA_27_-*b*-PS_130_) was synthesized through quaternization reactions between the BCP and methyl iodide [40,41]. The quaternization degree of the PDMAEMA_27_ block was around 50%. The TEM image of the copper particles used in this study is shown in Figure S2. The average size of the particles was determined to be around 400 nm.

**Figure 1:**
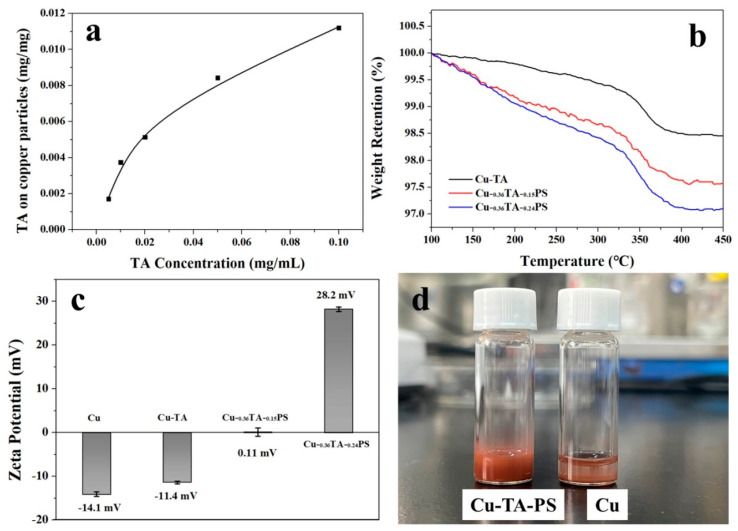
(**a**) The amounts of TA that is adsorbed on the surfaces of copper particles at different concentrations of TA; (**b**) thermogravimetric analysis (TGA) curves of Cu-TA, Cu-_0.36_TA-_0.15_PS and Cu-_0.36_TA-_0.24_PS; (**c**) zeta potential values of the copper particles, Cu-TA, Cu-_0.36_TA-_0.15_PS and Cu-_0.36_TA-_0.24_PS, and (**d**) photographs of copper and Cu-_0.36_TA-_0.24_PS particles in toluene.

After dispersing the copper particles in aqueous TA solutions of different concentrations, they were stirred for 10 h in the dark at room temperature. The amount of TA adsorbed on the surface of the copper particles was determined by UV–vis. The absorption spectra of TA at different concentrations and a standard curve are shown in Figure S3. We investigated the effect of the initial TA concentration on adsorption. In the adsorption tests, the concentration of copper particles was set at 10 mg/mL. The amount of TA adsorbed onto the copper particles at different TA concentrations was determined based on the standard curve. As shown in Figure 1a, the adsorption of TA on the surface of copper particles increased linearly, with the initial concentration at low concentrations, with a turning point at a concentration of 0.018 mg/mL. Above the critical concentration, the increase in the amount of adsorbed TA on the copper particles at this TA concentration is not as big as that in the low-concentration regime. When the initial concentrations of TA are 0.0025, 0.0050, 0.010 and 0.020 mg/mL, the surface densities of TA on the copper particles are calculated to be 0.36, 0.79, 1.08 and 2.36 molecules/nm^2^, respectively. Copper particles with a 0.36 molecules/nm^2^ surface density of TA are denoted as Cu-_0.36_TA. The radius of the projected area of a TA molecule is reported to be approximately 0.8 nm [42]; thus, the percentage of TA coverage on the Cu-_0.36_TA surface can be estimated to be about 76%. In this study, Cu-_0.36_TA was used in the synthesis of polymer brushes and surface nanostructures.

To prepare polymer brushes, qPDMAEMA_27_-*b*-PS_130_ was dissolved in DMF, and Cu-_0.36_TA particles were added in the polymer solution under stirring. After centrifugation, copper particles with polymer PS brushes on the TA coatings were obtained (Cu-_0.36_TA-PS). On the surfaces of the particles, qPDMAEMA_27_ blocks were grafted onto the TA coatings through electrostatic interactions and hydrogen bonding, and PS brushes on Cu-_0.36_TA were prepared (Scheme 1). It is reasonable to expect that the initial concentration of qPDMAEMA_27_-*b*-PS_130_ exerts a significant effect on the grafting density of PS brushes. Herein, polymer brushes were prepared at two different BCP concentrations, and thermogravimetric analysis (TGA) curves of Cu-_0.36_TA and the two different copper particles with PS brushes are shown in Figure 1b. The Cu-_0.36_TA particles have a total weight loss of 1.5 wt% in the range of 100–450 °C, and the two different particles with PS brushes have 2.25 and 2.88 wt% weight losses, respectively. Based on the TGA results, the surface densities of PS brushes on Cu-_0.36_TA particles are calculated to be 0.15 and 0.24 chains/nm^2^, respectively. The copper particles with two different PS surface densities are denoted as Cu-_0.36_TA-_0.15_PS and Cu-_0.36_TA-_0.24_PS, respectively.

The surface modification of copper particles by grafting qPDMAEMA_27_-*b*-PS_130_ results in large changes in the surface charges. The zeta potential values of the native copper particles, Cu-_0.36_TA, Cu-_0.36_TA-_0.15_PS and Cu-_0.36_TA-_0.24_PS, are shown in Figure 1c. The zeta potential of the native copper particles is -14.1 mV, and it changes to -11.4 mV after the coating of TA molecules on the surface (Cu-_0.36_TA). Upon the adsorption of the quaternized BCP chains, the zeta potentials of Cu-_0.36_TA-_0.15_PS and Cu-_0.36_TA-_0.24_PS increase to 0.1 and 28.2 mV due to the positively charged qPDMAEMA blocks on the particle surfaces. The grafting of polymer brushes significantly improves the dispersion of copper particles in organic solvents. As shown in Figure 1d, upon dispersion of the native copper particles in toluene, the particles precipitate from the solution immediately; in contrast, Cu-_0.36_TA-_0.24_PS particles can disperse in toluene for a while.

### 3.2. Construction of Surface Nanostructures on TA-Coated Copper Particles

In previous studies, it was reported that polymer brushes bonded covalently can be used to construct surface nanostructures by using surface co-assembly methods, and a variety of surface nanostructures including spherical surface micelles (s-micelles), worm-like structures and layered surface structures are formed. Herein, surface co-assembly of Cu-_0.36_TA-_0.15_PS (or Cu-_0.36_TA-_0.24_PS) and PDMAEMA_50_-*b*-PS_136_ at different BCP concentrations was performed in a THF/methanol mixture. To perform surface co-assembly, 10 mg of Cu-_0.36_TA-_0.15_PS (or Cu-_0.36_TA-_0.24_PS) was dispersed in 1.0 mL of THF, and varying amounts of PDMAEMA_50_-*b*-PS_136_ was dissolved in the solution. Excess methanol (7.0 mL) was added in a dropwise manner to the mixture under stirring. Copper particles with surface nanostructures were collected after centrifugation. As shown in Scheme 1, upon the addition of methanol, the PS brushes on the surface of the copper particles and the PS blocks in the BCP collapsed to form the core of the surface nanostructures, and the PDEMA blocks stabilized the nanostructures by stretching outwards.

**Figure 2:**
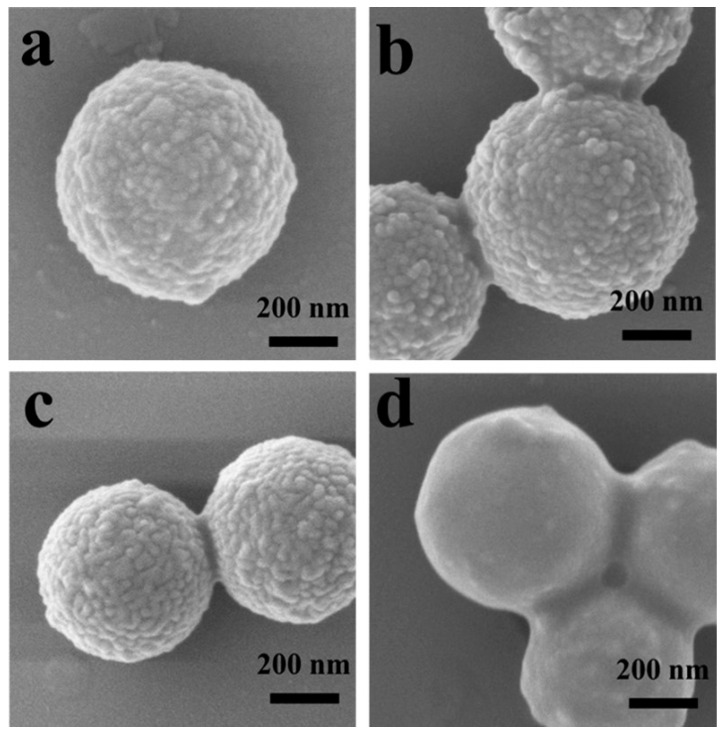
SEM images of surface nanostructures on TA-coated copper particles formed through surface co-assembly of Cu-_0.36_TA-_0.15_PS and PDMAEMA_50_-*b*-PS_136_ at BCP concentrations of (**a**) 0.25, (**b**) 0.37, (**c**) 0.50 and (**d**) 0.63 mg/mL. To fabricate surface nanostructures, Cu-_0.36_TA-_0.15_PS and different amounts of PDMAEMA_50_-*b*-PS_136_ were dissolved in THF and seven-fold volumes of methanol were added into solutions.

Figure 2 shows the SEM images of surface structures formed by Cu-_0.36_TA-_0.15_PS and PDMAEMA_50_-*b*-PS_136_ at different BCP concentrations. Spherical s-micelles are formed at BCP concentrations of 0.25, 0.37 and 0.50 mg/mL, and layered structures are formed at 0.63 mg/mL, indicating that s-micelles are formed at low BCP concentrations and layered nanostructures are formed at high BCP concentrations. It is noted that at 0.50 mg/mL, short worm-like s-micelles are also observed (Figure 2c). The formation of worm-like structures is attributed to the fusion of the neighboring isolated s-micelles formed at a high BCP concentrations. In a previous study, it was reported that with an increase in BCP concentration, the surface morphology experienced a change from isolated s-micelles to large-sized s-micelles, then to worm-like structures and finally to layered nanostructures [34]. Herein, with an increase in BCP concentration, more BCP chains are involved in the surface co-assembly; and in order to accommodate “free” BCP chains in the surface co-assembly process, short worm-like s-micelles and layered nanostructures are formed at high BCP concentrations.

**Figure 3:**
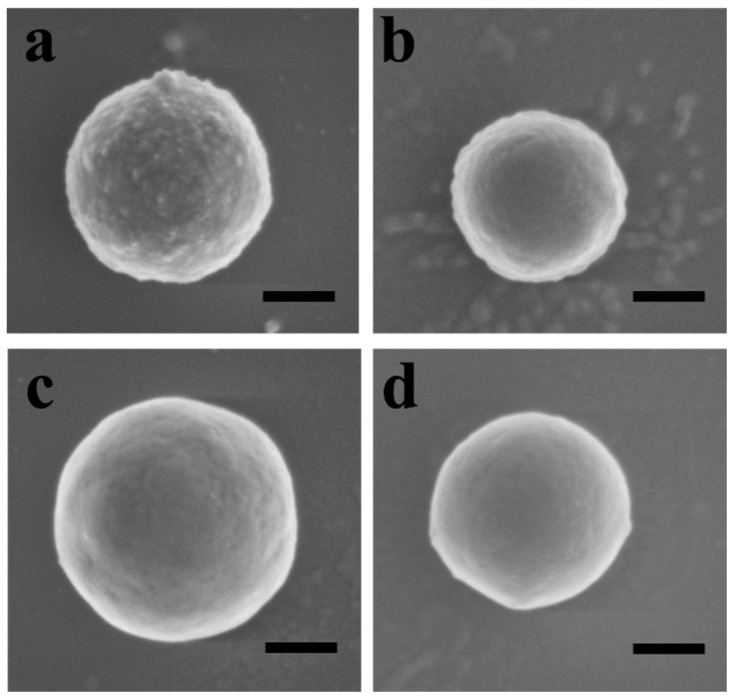
SEM images of surface nanostructures on TA-coated copper particles formed through surface co-assembly of Cu-_0.36_TA-_0.24_PS and PDMAEMA_50_-*b*-PS_136_ at BCP concentrations of (**a**) 0.13, (**b**) 0.25, (**c**) 0.63 and (**d**) 1.2 mg/mL. Scale bars in images represent 200 nm.

The SEM images of surface nanostructures formed through the co-assembly of Cu-_0.36_TA-_0.24_PS and PDMAEMA_50_-*b*-PS_136_ at different BCP concentrations are shown in Figure 3. At 0.13 mg/mL, spherical s-micelles are observed on the copper particles, and layered structures are formed at a BCP concentration above 0.25 mg/mL. In comparison with Cu-_0.36_TA-_0.15_PS, the formation of layered structures on Cu-_0.36_TA-_0.24_PS is favored at the same BCP concentration. The grafting density of PS plays an important role in the fabrication of surface nanostructures. During surface co-assembly, PS brushes co-collapse with PS blocks to form the core of the s-micelles, and PDMAEMA blocks stabilize the nanostructures. As the grafting density of PS brushes increases, more BCP chains are required to stabilize the nanostructure during surface co-assembly. The layered nanostructures are able to accommodate more BCP chains than the spherical s-micelles, so the formation of the layered structures is favored on Cu-_0.36_TA-_0.24_PS at the same concentration of BCP.

**Figure 4:**
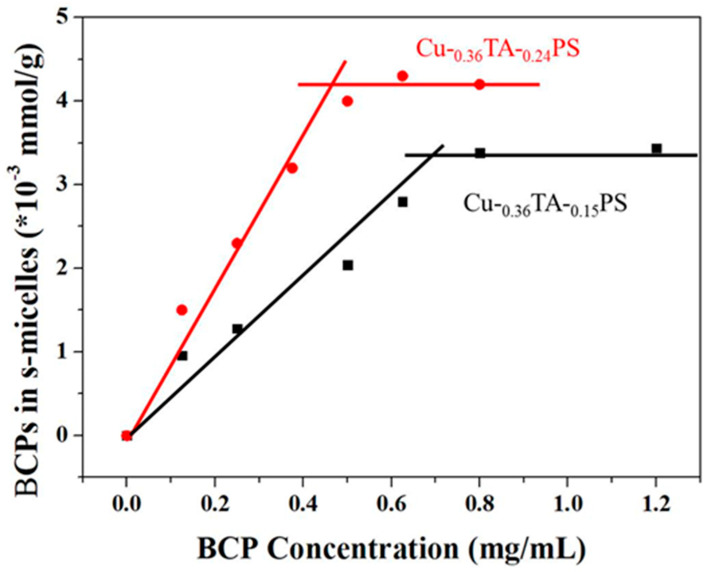
Amounts of PDMAEMA_50_-*b*-PS_136_ BCP involved in surface co-assemblies with Cu-_0.36_TA-_0.15_PS and Cu-_0.36_TA_-0.24_PS at different BCP concentrations.

In order to determine the amount of BCP chains taking part in surface co-assembly, the copper particles with surface nanostructures were dispersed in DMF, and the BCP chains in the nanostructures were released into the solution. The “free” BCP contents in the solution were determined by UV–vis after centrifugation of the copper particles. The absorption spectra of BCP at different concentrations and a standard curve are shown in Figure S4. Figure 4 shows the contents of free BCP chains in the nanostructures on Cu-_0.36_TA-_0.15_PS and Cu-_0.36_TA-_0.24_PS formed at different BCP concentrations. Because of the higher grafting density of polymer brushes on the particles, at the same BCP concentration, more BCP chains are involved in the surface co-assembly on Cu-_0.36_TA-_0.24_PS than on Cu-_0.36_TA-_0.15_PS. On both plots, the content of BCP in the nanostructures increases with low BCP concentrations and remains constant after reaching a critical value. The amount of BCP chains involved in the surface self-assembly on the copper particles is related to the surface ratio of the particles and the grafting density of PS. A higher grafting density of PS is favorable for the adsorption of BCP chains onto the particle surfaces. The critical values on the plots represent the saturation concentrations of the BCP chains on the surfaces, and no more BCP chains take part in the surface co-assembly above the critical values.

### 3.3. Synthesis of Hollow Capsules

**Figure 5:**
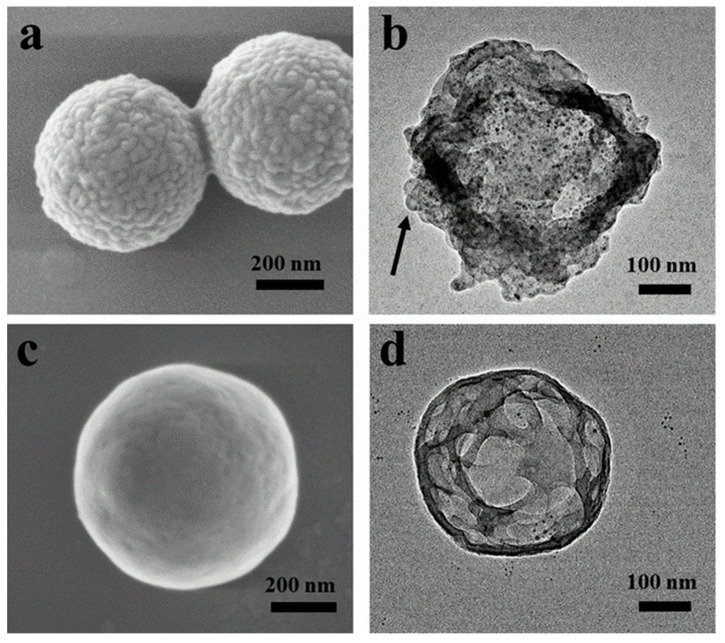
Copper particles with surface nanostructures self-assembled by PS brushes on Cu-_0.36_TA-_0.15_PS particles and PDMAEMA_50_-*b*-PS_136_ before (**a**) and after (**b**) etching with hydrochloric acid, and copper particles with surface nanostructures self-assembled by PS brushes on Cu-_0.36_TA-_0.24_PS particles and PDMAEMA_50_-*b*-PS_136_ before (**c**) and after (**d**) etching with hydrochloric acid. Images (**a**,**c**) are SEM images and (**b**,**d**) are TEM images.

Inorganic particles with polymer coatings on the surfaces can be used as templates for the synthesis of hollow capsules. Herein, Cu-_0.36_TA-_0.15_PS and Cu-_0.36_TA-_0.24_PS particles with surface nanostructures are used as templates and hollow capsules are prepared by etching the copper particles with hydrochloric acid. Figure 5a shows the SEM images of copper particles with surface nanostructures self-assembled by PS brushes on Cu-_0.36_TA-_0.15_PS particles and PDMAEMA_50_-*b*-PS_136_. On the copper surfaces, spherical s-micelles formed by PS brushes and PDMAEMA_50_-*b*-PS_136_ BCP chains are observed. Copper particles were etched with HCl acid and hollow capsules were obtained. As indicated by an arrow, s-micelles are located at the surfaces of the hollow capsules (Figure 5b), which suggests that the surface structures are maintained in the etching process. Figure 5c represents the SEM images of copper particles with surface nanostructures self-assembled by PS brushes and PDMAEMA_50_-*b*-PS_136_ on Cu-_0.36_TA-_0.24_PS particles. Layered nanostructures are formed on the surfaces of the particles. After etching the copper particles, hollow capsules are formed (Figure 5d). TA molecules form coatings on the surfaces of the copper particles. Upon etching the copper particles with HCl acid, copper ions are produced, which form complexes with TA molecules on the coatings and result in the formation of the cross-linked coatings. The surface of the hollow capsules is composed of the surface nanostructures, spherical s-micelles or layered nanostructures, which are anchored to the TA coatings through the electrostatic interaction.

## 4. Conclusions

TA molecules can be adsorbed onto copper particles, which leads to the formation of TA coatings. PS brushes can be synthesized on the TA coatings through electrostatic interactions and hydrogen bonding between qPDMAEMA_27_-*b*-PS_130_ and the TA coatings. The PS brushes on the copper surfaces are able to achieve surface co-assembly with “free” BCP chains and surface nanostructures are fabricated on the copper particles. The PS grafting density exerts influence on the amount of BCP in the surface nanostructures and on the surface morphology. After etching the copper particles with HCl acid, hollow capsules are obtained. The surface nanostructures on the particles are maintained in the etching process. The copper particles with PS brushes will find applications for many different purposes. This study provides a versatile method for the synthesis of polymer brushes on metal surfaces. It is reasonable to expect that the TA-coating approach will find applications in achieving the anti-corrosion of metals.

## Data Availability

The raw data supporting the conclusions of this article will be made available by the authors on request.

## Supplementary Materials

The following supporting information can be downloaded at: https://www.mdpi.com/article/10.3390/polym16111587/s1, Figure S1: (a) ^1^H NMR spectrum of PDMAEMA_27_-*b*-PS_130_ and (b) size exclusion chromatography (SEC) curves of PDMAEMA_50_-*b*-PS_136_, PDMAEMA_27_-*b*-PS_130_ and their precursors; Figure S2: TEM image of commercial copper particles; Figure S3: (a) Absorption of TA at different concentrations, (b) standard curve of absorption vs. TA concentration; Figure S4: (a) Absorption of PDMAEMA_50_-*b*-PS_136_ BCP at different concentrations, (b) standard curve of absorption vs. BCP concentration.
