# Supplementary material for: Synthesis of Polymer Brushes on Tannic Acid-Coated Copper Particles and Surface Co-Assembly
Source: Polymers (Basel). 2024 Jun 3;16(11):1587. doi: 10.3390/polym16111587 (PMC11175133; doi:10.3390/polym16111587)
Supplement: Supplementary file 1 [file polymers-16-01587-s001.zip › polymers-2957284-supplementary.pdf]

Supporting Information

Synthesis of Polymer Brushes on Tannic Acid-Coated Copper Particles and Surface Co-Assembly

Chen Wang and Hanying Zhao

College of Chemistry and Key Laboratory of Functional Polymer Materials of the Ministry of Education, Nankai University, Tianjin 300071, China

CONCENTS

1. SUPPLEMENTARY FIGURES 2

Figure S1. 2

Figure S2. 3

Figure S3. 3

Figure S4. 4

1. SUPPLEMENTARY FIGURES

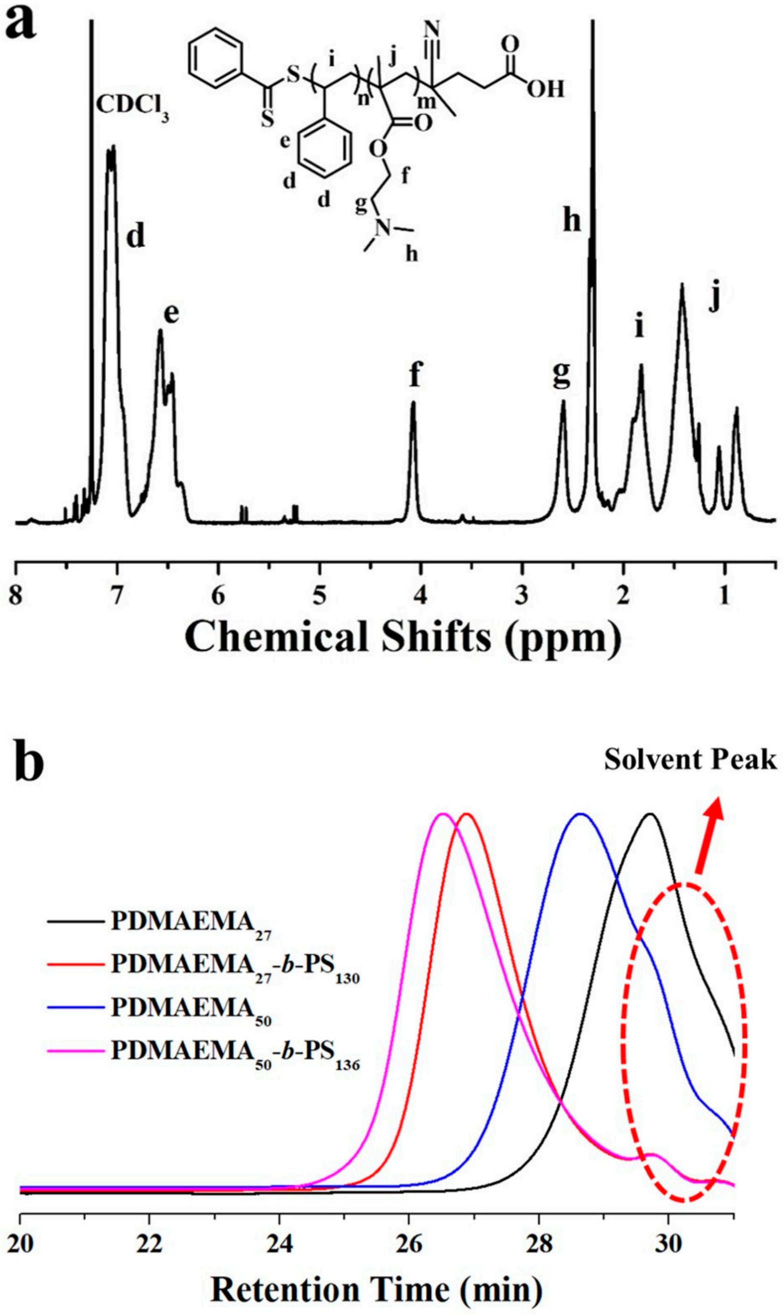

Figure S1. (a) ^{1}H NMR spectrum of PDMAEMA$_{27}$-*b*-PS$_{130}$ and (b) size exclusion chromatography (SEC) curves of PDMAEMA$_{50}$-*b*-PS$_{136}$, PDMAEMA$_{27}$-*b*-PS$_{130}$ and their precursors.

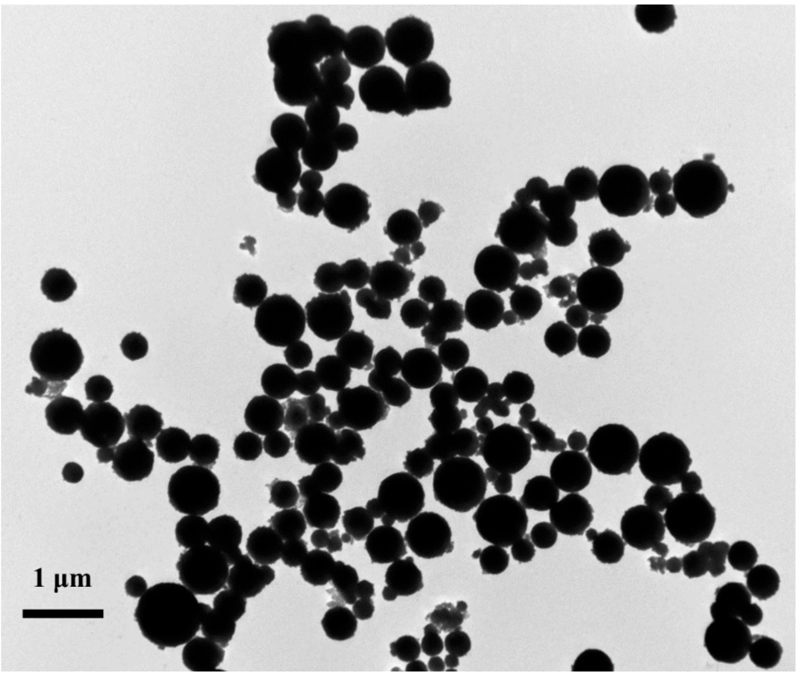

Figure S2. TEM image of commercial copper particles.

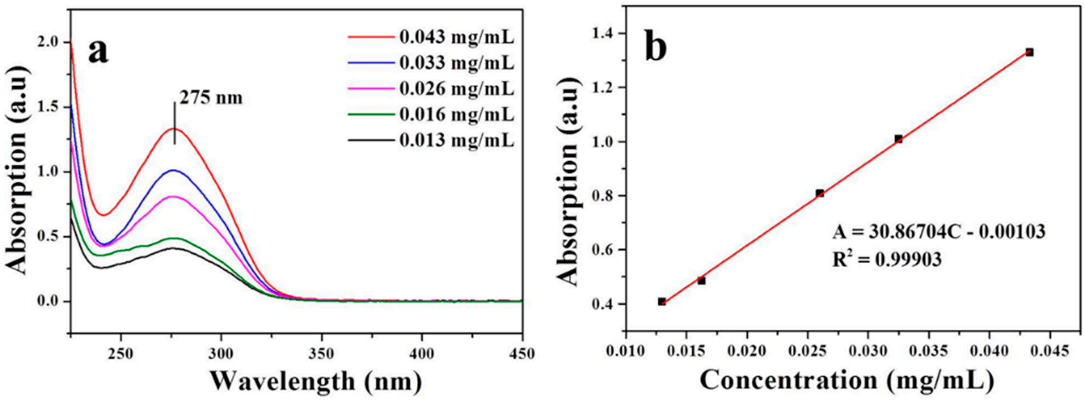

Figure S3. (a) Absorption of TA at different concentrations, (b) a standard curve of absorption *vs.* TA concentration.

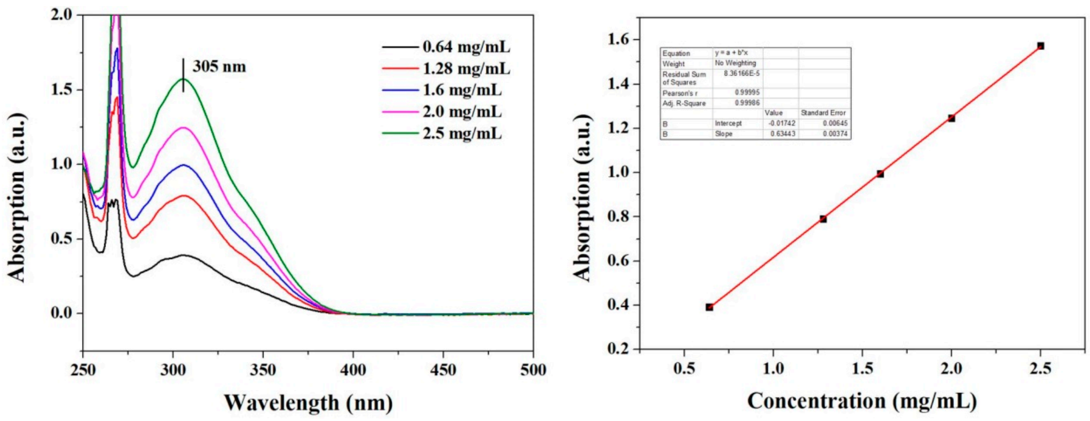

Figure S4. (a) Absorption of $PDMAEMA_{50}$-b-PS_{136} BCP at different concentrations, (b) a standard curve of absorption *vs.* BCP concentration.
